# Endothelial Basement Membrane Components and Their Products, Matrikines: Active Drivers of Pulmonary Hypertension?

**DOI:** 10.3390/cells9092029

**Published:** 2020-09-03

**Authors:** Ayse Ceren Mutgan, Katharina Jandl, Grazyna Kwapiszewska

**Affiliations:** 1 Otto Loewi Research Center, Division of Physiology, Medical University of Graz, 8010 Graz, Austria; ayse.mutgan@medunigraz.at; 2 Ludwig Boltzmann Institute for Lung Vascular Research, 8010 Graz, Austria; katharina.jandl@lvr.lbg.ac.at; 3 Otto Loewi Research Center, Division of Pharmacology, Medical University of Graz, 8010 Graz, Austria

**Keywords:** IPAH, vascular remodeling, basement membrane, laminin, type IV collagen, matrikines, endostatin, BMPRII, barrier function, apoptosis

## Abstract

Pulmonary arterial hypertension (PAH) is a vascular disease that is characterized by elevated pulmonary arterial pressure (PAP) due to progressive vascular remodeling. Extracellular matrix (ECM) deposition in pulmonary arteries (PA) is one of the key features of vascular remodeling. Emerging evidence indicates that the basement membrane (BM), a specialized cluster of ECM proteins underlying the endothelium, may be actively involved in the progression of vascular remodeling. The BM and its steady turnover are pivotal for maintaining appropriate vascular functions. However, the pathologically elevated turnover of BM components leads to an increased release of biologically active short fragments, which are called matrikines. Both BM components and their matrikines can interfere with pivotal biological processes, such as survival, proliferation, adhesion, and migration and thus may actively contribute to endothelial dysfunction. Therefore, in this review, we summarize the emerging role of the BM and its matrikines on the vascular endothelium and further discuss its implications on lung vascular remodeling in pulmonary hypertension.

## 1. Introduction

Pulmonary arterial hypertension (PAH) is a rare but severe pulmonary vascular disease with poor prognosis and survival. It is characterized by elevated mean pulmonary arterial pressure (mPAP) resulting in right ventricle hypertrophy and right heart failure [1,2]. Aberrant and progressive pulmonary vascular remodeling with lumen obstruction and vessel wall thickening in both proximal and distal pulmonary arteries (PAs) underlies the pathological features [2,3,4]. Remodeling is observed in all three layers of the vessel wall: the outer adventitia layer (marked by platelet derived growth factor receptor alpha (PDGFRα) positive cells), the middle media, and the neointima layer (marked by alpha smooth muscle actin (αSMA) positive cells) [5,6]. Although both smooth muscle cells (SMCs) and fibroblasts expand during vascular remodeling processes, endothelial cells (ECs) control the vascular tone and SMC proliferation by secreting vasoactive and proliferative factors such as platelet-derived growth factor-BB (PDGF-BB) and endothelin-1 (ET-1) [7,8,9]. ECs also regulate the vascular wall integrity and form a tight barrier, thereby controlling immune cell adhesion and transmigration [10]. Hence, pathological changes leading to endothelial dysfunction can significantly contribute to the pathophysiology and progression of cardiovascular diseases, including pulmonary hypertension (PH) [11,12,13,14].

All ECs are underlined by the basement membrane (BM), a specialized extracellular matrix (ECM), which is compulsory for the structural integrity and stability of blood vessels. The major BM components are laminins, type IV collagens, and BM proteoglycans (BM heparan sulfate proteoglycans and other BM glycoproteins) [15]. In addition to their structural function, accumulating evidence indicates that BM proteins actively modulate cellular processes. This is accomplished in multiple ways: First, all major components can directly interact with cells via specific cell surface receptors, thereby influencing the cell shape, cell motility, proliferation, and intracellular signaling programs [16,17]. Second, the BM alters cell function by acting as a reservoir for growth factors and morphogens [18,19], such as fibroblast growth factor (FGF) [20], transforming growth factor β (TGF-β) [21], and vascular endothelial growth factor (VEGF) [22]. Third, biologically active short fragments of ECM proteins, named matrikines, can be released from the BM and actively alter EC behavior and function independent from their parent molecule [23].

Changes in the quantity, composition, and structure of BM are often associated with vasculopathies, and well documented in a variety of systemic diseases, such as diabetes [24], atherosclerosis [25], or neurological disorders, such as Alzheimer’s disease [26]. Exemplarily, capillary BM thickening is one of the hallmarks of long-term diabetes, and it likely arises due to the increased expression of BM components, leading to diabetic retinopathy and loss of vascular elasticity, and thereby contributing to elevated blood pressure [27,28].

Elevated expression of BM components is often accompanied by increased BM degradation. BM degradation is a prerequisite for transendothelial immune cell migration, and it is therefore associated with the accumulation of inflammatory cells, as observed in neurodegenerative diseases including stroke [26], multiple sclerosis [29], and in cancer [30]. As a consequence of BM degradation, biologically active matrikines might be liberated, which further propagate pathological effects [31,32,33]. In multiple cases, matrikines levels have been correlating with disease severity [34,35] and thus can serve as biomarkers [34,35,36,37,38,39].

Similarly, in lung diseases with vascular remodeling and pulmonary hypertension, alterations in the BM [4,5] and the release of matrikines have been described [5,40,41]. However, in comparison to systemic disorders, the role of the BM is still under-investigated in this field. In this review, we will use the vast knowledge of the BM on endothelial function in general, summarize the accumulating evidence of the BM involvement in pulmonary vascular remodeling, and provide an outlook for future investigations in the PH field.

## 2. General Function of BM Components on the Vascular Endothelium

The thickness of the BM varies from tissue to tissue, and ranges from 50 to 500 nm [42]. On an ultrastructural level, the BM is composed of two layers: lamina lucida and lamina densa, which are made up by two individual networks of laminin (lucida) and type IV collagen (densa) connected by BM proteoglycans [15,43]. They assemble in a sophisticated yet simple structure. In brief, laminin binding to its receptors on the basal side initiates a concentration-induced polymerization process [42,44]. Type IV collagen binds to laminin and its polymerization forms the second network. Nidogens and proteoglycans such as perlecan and agrin stabilize the two networks. Nidogens bind both to laminin and type IV collagen, forming a non-covalent high-affinity stabilizing bridge. Then, agrin and perlecan further connect nidogens and laminin to support the formation and stability of the BM [42]. All major structural components (type IV collagen, laminins, and proteoglycans) can be glycosylated and are therefore classified as glycoproteins [45,46]. For a fully functional BM, all components are needed. However, each class and subtype individually confer unique features, which will be discussed in the next paragraph and are summarized in Table 1.

### 2.1. BM Type IV Collagens

Type IV collagen is the most abundant constituent of the BM [47]. Six genetically distinct type IV collagen chains (α1–6 chain) are encoded by COL4A1–A6 [48]. All chains are highly homologous and contain three structurally distinct domains: an amino-terminal domain rich in cysteine and lysine residues, a major collagenous domain, and a carboxyl-terminal non-collagenous (NC1) domain [48]. In each chain, the C-terminal NC1 domain is unique [49]. Type IV collagen chains remarkably assemble into only three distinct heterotrimers: α1α1α2, α3α4α5, and α5α5α6 [47]. The type IV collagen α1α1α2 heterotrimers are ubiquitously found in all BMs, whereas α3α4α5 trimers are more tissue specific and α5α5α6 trimers are often found as a pericellular BM of mesenchymal cells [48]. While a rudimentary BM can be formed without type IV collagen, its deletion is still detrimental [50,51]. In vivo, homozygous knockout of Col4a1 and Col4a2 (Col4a1^−/−^; Col4a2^−/−^) is embryonically lethal due to disorganized angiogenesis during the development of capillary networks [50]. Heterozygous mutants often die soon after birth due to cerebral hemorrhages and respiratory distress [52,53]. Similarly, in patients, mutations in COL4A1/COL4A2 often manifest in vascular defects in the brain concomitant with hemorrhages in the lung, kidney, and retina [51], and mutations in COL4A3-5, clinically, lead to Alport’s Syndrome, which is characterized by renal failure and alveolar hemorrhages [54]. Hence, type IV collagen mutations mostly affect tissues that are exposed to increased mechanical stress, such as the heart, blood vessels, or kidney, highlighting its importance in vessel stability.

All type IV collagen heterotrimers are stabilized by covalent disulfide bonds at varying degrees, with α3α4α5 heterotrimers showing a comparably high degree of crosslinking [55]. This likely enables them to resist increased stress (mechanical, chemical, enzymatical) in the healthy vasculature [56]. Type IV collagens directly bind to cell surface receptors, such as integrins or discoidin domain receptors, and initiate signaling pathways that are often associated with proliferation, migration, and polarity in order to sustain vascular stability [47,48].

### 2.2. BM-Laminin

Laminins (LNs) are the most important component of the BM [57,58,59] and are indispensable for BM formation [57]. By the combination of independent α β and γ chains, 16 different LN heterotrimers with a common cruciform-like structure can be formed and LN isoform expression is often tissue and time specific [60]. Generally, all vessels are enriched for LN α4 and α5 chain [61] incorporated into the two major endothelial BM LNs LN-411 (LNα4β1γ1) and LN-511 (LNα5β1γ1) [61,62,63,64]. LN-411 is expressed ubiquitously in all types of vessels, while the expression pattern of LN-511 varies in different vessels [62] and tissues, with higher levels in the placenta, lung, and pancreas [65].

In vivo, an absence of LN-α4 chain (Lama4^−/−^) leads to leakages in the microvasculature [58]. Initiation of expression of Lama5 (LN-α5) at later stages in development, can partially take over the function of Lama4 and to some extent inhibit the microleakages [58]. Thus, both LN-α4 and α5 chains seem to confer BM stability and barrier integrity. Interestingly, immune cell transmigration is more prominent on spots with lower LN-511 abundancy [66,67,68], and an absence of LN-511 in vivo results in thickened vessels that are unable to dilate in response to shear stress [69]. Thus, the LN-α5 chain additionally contributes to the regulation of vessel tone and immune cell transmigration.

In the adult vessel, the preferential β chain is the LN-β1 chain. In younger and/or developing vessels, LN-β2 chain is expressed also, forming LN-421 and LN-521 [70]. Similar to the α chain, individual β and γ chains also mediate differential EC responses. For instance, when exposed to LN-421 compared to LN-411, ECs show a more activated phenotype, which is recapitulated by increased adhesion, migration, tube formation, and the secretion of thrombospondin-2 and FGF [70]. This suggests that the β chain is involved in mediating EC integrity and stability.

Amongst all γ chains, LN–γ encoded by LAMC1 is the most abundantly expressed one. An absence of Lamc1 is embryonically lethal and laminin sheet formation cannot be completed, indicating that LN-γ1 is absolutely essential for the formation and assembly of the BM [57].

### 2.3. BM-Nidogens

Other predominant BM components are nidogens, NID-1 and NID-2, belonging to the glycoprotein family. Nidogens are responsible for the establishment of ternary complexes by interacting with laminin, type IV collagens, fibulin, perlecan, and type XVIII collagen α1 [71,72,73,74,75]. NID-1 is ubiquitously expressed in all vessel layers, while NID-2 is mostly enriched in endothelial BM [76]. NID-1 and NID-2 can partially compensate each other’s function as in absence of either of the NID isoforms, the BM can still assemble [76,77,78], however, with limited barrier integrity [79]. On the contrary, the absence of both nidogen isoforms in the BM leads to impaired BM in capillaries [80] and perinatal lethality [81].

### 2.4. BM Heparan Sulfate Proteoglycans: Perlecan, Type XVIII Collagen α1, and Agrin

Heparan sulfate proteoglycans (HSPGs) consist of a protein core attached to long linear glycosaminoglycan heparan sulfate chains [82]. The most prominent members of HSPGs in the BM are perlecan, agrin, and type XVIII collagen α1 [18]. HSPGs provide a structural support to BM, facilitate cell–cell and cell–matrix interactions (by binding to integrins and adhesion receptors), cell motility, and cell migration [83]. Within their glycosaminoglycan chains, bioactive ligands such as chemokines, cytokines, morphogens, and growth factors can be tethered, and gradients can be established [18,84]. Thereby, they can modulate the activity of cellular signaling pathways, such as tyrosine receptor kinase related signaling [85]. Pro-inflammatory cytokines such as tumor necrosis factor-alpha (TNF-α), interleukin-1 alpha (IL-1α), or interleukin-1 beta (IL-1β) affect the expression of HSPGs and thus, their relative contribution in the BM [86]. Similar to other BM components, the BM HSPG expression and composition is tissue-specific.

Perlecan is the major HSPG in the vascular BM. Knockout studies showed that perlecan is crucial to maintain BM integrity and important for vasculogenesis [87]. The adhesion of EC to perlecan increased endothelial barrier function [88] and augmented cell growth [89,90].

Type XVIII collagen α1 was the first collagen discovered to carry heparan sulfate (HS) side chains [91]. Type XVIII collagen α1 has three isoforms (short, medium, and long) [92]. In the vasculature, the short isoform of type XVIII collagen α1 is predominant [92,93,94]. Type XVIII collagen α1 is important for BM integrity as COL18A1 knockout is associated with structural changes in BM and enhanced vascular permeability in vivo [95].

Agrin is another major vascular BM-HSPG [96]. In vitro, adhesion to agrin augmented endothelial barrier integrity [88] possibly via enhancing VE-Cadherin, β-catenin, and zonula ocluddin-1 (ZO-1) at the EC junctions [97]. Even though current studies on the function of agrin in vascular endothelial BM are still scarce, accumulating data indicates that similarly to other BM-HSPGs, agrin is important for BM integrity and stability.

After providing the overview of how the individual BM component can alter EC function, the next section will describe the role of BM fragments, matrikines, on EC behavior.

## 3. BM Matrikines

Matrikines are proteolytic fragments of ECM proteins with a biological function distinct to that of their parent molecule [23]. Although the BM is rather insoluble [98], stable, and resistant to external factors, it is not static. Indeed, a constant cycle of generation and degradation of BM proteins is a biological process that is necessary to maintain a tissue homeostasis [99]. Even in its physiological state, the BM harbors and sequesters BM degrading enzymes such as disintegrin and metalloproteases (ADAMs), matrix metalloproteinases (MMPs), serine proteinases (such as cathepsins), and their inhibitors (e.g., tissue inhibitors of metalloproteinases (TIMPs) [23,100]. However, in disease, an imbalance in production and degradation can lead to the increased fragmentation of BM proteins, resulting in the pronounced release of matrikines. In turn, these matrikines can then actively modulate cellular functions.

The most thoroughly investigated BM matrikines are derived from type IV collagen, type XVIII collagen α1, and perlecan. A list of all BM-derived matrikines can be found in Table 2. As their naming suggests (-statins), BM matrikines often have limiting functions on EC, including the inhibition of angiogenesis, migration, or proliferation [101,102,103,104], which will be discussed in detail in the following section.

### Regulation of EC Function by Matrikines

Angiogenesis is the formation and growth of new blood vessels from pre-existing vessels [138]. The underlying processes are EC proliferation and migration, which are mediated by a balance of pro-angiogenic and anti-angiogenic factors [139]. Increased angiogenesis is a hallmark of cancer [140] and the anti-angiogenic function of several BM matrikines harbors a huge potential for anti-cancer therapy [141]. An excellent review is available for matrikines in cancer [30]; therefore, this section will only briefly touch upon this subject. Besides cancer, dysbalances in pro- and anti-angiogenic factors are implicated in the pathogenesis of a variety of other, non-cancerous diseases, including lung diseases, such as idiopathic pulmonary fibrosis (IPF) [142], chronic obstructive pulmonary disease (COPD) [143], or lung vascular diseases such as PH [5,41].

Tube formation and the biologically more complex chicken chorioallantoic membrane model (CAM) are often used to measure and quantify angiogenesis in experimental settings [138]. Type IV collagen matrikines arresten (α1), canstatin (α2), and tumstatin (α3) were able to inhibit tube formation [112,144,145,146] and the NC1 domain of type IV collagen α6 potently inhibited the FGF-mediated angiogenesis in the chicken CAM model [113]. In contrast, the full-length NC1 domains of type IV collagen α4 and α5 did not affect angiogenesis in the chicken CAM model. However, they potently limited EC adhesion and migration in vitro [113] and the type IV collagen α5 NC1 domain additionally inhibited lymphangiogenesis [119]. Recently, the biologically active sequences within the NC1 domains of type IV collagen α4, α5, and α6 chains were identified in bioinformatics approaches and named as tetrastatin, pentastatin, and hexastatin, respectively [118]. Those 20 amino acid-long peptides indeed possessed anti-proliferative and anti-migratory effects in vitro and in vivo comparable to their parents NC1 domains [117,147,148]. However, for most of the type IV collagen α4, α5, and α6 derived matrikines, the cellular mechanisms behind the effect are yet to be identified. The major HSPG-derived matrikines, endostatin (type XVIII collagen α1) and endorepellin (perlecan), inhibited angiogenesis as measured by reduced endothelial migration, tube formation, and blood vessel growth in and ex vivo [121,149]. Part of their action is mediated by the blocking of pro-angiogenic signaling pathways. Endostatin and endorepellin conduct their anti-angiogenetic function on EC via interfering with VEGF signaling. Endostatin antagonizes vascular endothelial growth factor receptor 2 (VEGFR2) signaling by direct binding to VEGFR2 [130], while endorepellin diminishes VEGFR downstream signaling by dual receptor binding to VEGFR2 and α2β1 integrin [123,124,150,151].

In addition, many BM matrikines have been reported to initiate apoptosis via a variety of pathways. The Fas Ligand (FasL) is one of the primary extrinsic apoptosis pathways [152] as well as a target for BM arresten and canstatin. Arresten, canstatin, and tumstatin also initiate apoptosis by disruption of focal adhesion kinase/phosphoinositide-3 kinase (FAK/PI3K) signaling [114,153,154,155], which is a pathway normally involved in cell anchorage and motility [156]. Additionally, matrikines can abolish protein synthesis in EC via inhibition of the mammalian target of rapamycin (mTOR) pathway, as described for tumstatin [153] and canstatin [114]. A reduction in protein synthesis has been associated with the autophagy of cells [157], and endostatin and endorepellin, matrikines of BM-HSPG, have been described to mediate autophagy via Beclin-1 [158,159,160]. Endostatin additionally acts on the small heterodimer partner (SHP) nuclear death receptor, which upon translocation to the mitochondria initiates apoptosis via B-cell lymphoma 2 (Bcl-2) [161,162].

**Figure 1:**
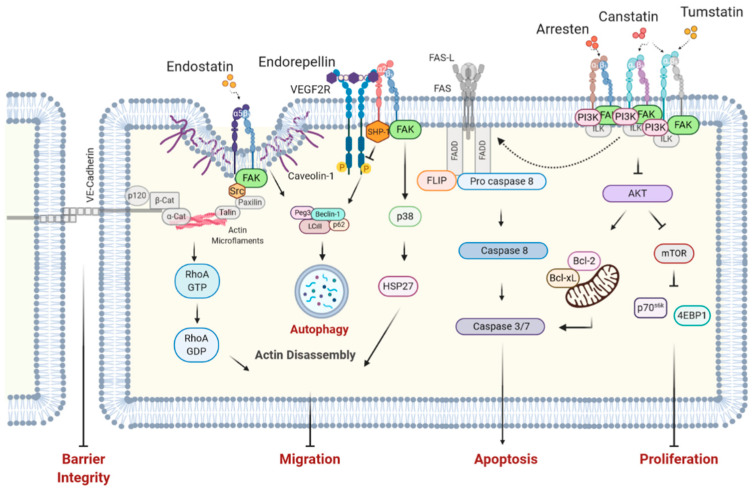
**Possible mode of action of matrikines on pulmonary arterial endothelial cells.** Type IV collagen matrikines alter cell survival in endothelial cells. Arresten and canstatin activate Fas-L-mediated apoptosis (caspase 8 activation) via an unknown mechanism [154,155]. Upon integrin binding, arresten, canstatin, and tumstatin induce caspase-9 dependent mitochondrial apoptosis via inhibiting FAK phosphorylation [114,153,154,166]. Canstatin and tumstatin attenuate proliferation by inhibiting the FAK/PI3K/AKT/mTOR signaling pathway [114,153]. BM HSPGs influence cell survival and cell motility. Endostatin and endorepellin activate autophagy in a Beclin1-dependent manner [158,159]. In addition, endostatin and endorepellin trigger the disassembly of actin stress fibers by inhibiting caveolin-associated Src-mediated RhoA kinase [164] and by activating the FAK/p38/HSP27 signaling pathway [165], respectively. Hence, matrikines can interfere with crucial EC functions involved in PH pathophysiology. Receptors and signaling proteins involved in the depicted pathways, but not directly investigated in the indicated studies, are represented in gray. Fas-L, Fas ligand; FAK, focal adhesion kinase; PI3K, Phosphoinositide 3-kinases; AKT, alpha serine/threonine-protein kinase; mTOR, mammalian target of rapamycin; BM HSPGs, basement membrane heparan sulfate proteoglycans; HSP27, heat shock protein 27; EC, endothelial cells; PH, pulmonary hypertension.

BM matrikines such as endostatin and endorepellin also interfere with EC cytoskeletal rearrangement. The binding of endostatin to α5β1 integrins and caveolin-1 inhibits focal adhesion complex, thereby leading to cytoskeletal disruption via Src-mediated downregulation of RhoA GTPase [129,163,164]. Endorepellin similarly induces actin disassembly by engaging with α2β1 integrins to activate cyclic adenosine monophosphate (cAMP)-dependent protein kinase A (PKA) and FAK [165]. By interfering with the cytoskeletal arrangement, HSPG matrikines potently inhibit EC migration. An overview of the functional effects mediated by matrikines on endothelial cells can be found in Table 3. Accordingly, possible mechanisms of action of matrikines in pulmonary arterial endothelial cells (PAECs) are depicted in Figure 1.

## 4. BM Components and Their Matrikines in PH

The individual components of the BM and its matrikines are potent modulators of EC function, interfering with proliferation, apoptosis, barrier integrity, and vasoreactivity, all of which have been implicated in the development and progression of PH. Thus, in the next section, we will summarize the current knowledge on the BM remodeling in pulmonary arteries and the possible involvement of matrikines in PH pathogenesis.

### 4.1. Changes in BM Ultrastructure and Composition in PH

The main feature of PH is the remodeling of distal and proximal PAs accompanied by increased ECM deposition and turnover [167]. This takes place in all three layers of the vessel wall, with the neointima being most affected [4,5,168]. Increasingly, research identified that ECM deposition is not merely a consequence, but an active driver of the disease [169]. Although most of the research so far has focused on interstitial, fibrillar collagens (type I and type III collagen) [169], increasingly, the BM and its components also emerge as critical factors in PH.

Already in 1974, a case report by the group of Lynne Reid described an increase in the thickness of the pulmonary capillary endothelial basement membrane on an ultrastructural level [170]. Accordingly, studies using animal models of PH also showed an accumulation of BM proteins, with increased expression of subendothelial BM proteins laminin, type IV collagen and perlecan (BM HSPG) [171,172]. Despite all these evidences, the BM remained under-investigated for many years; however, recently, it has reemerged as a promising research area in the field of PH.

**Figure 2:**
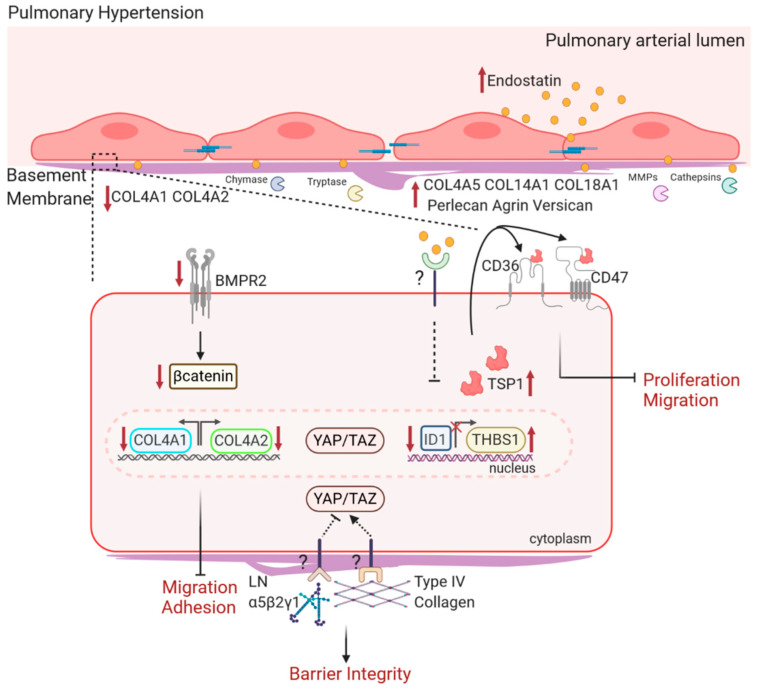
**Current knowledge on the role of basement membrane components in pulmonary endothelial cell function in IPAH.** In IPAH, the structure and composition of the BM is altered [4]. In PAs, COL4A5, COL14A1, COL18A1, perlecan, agrin, and versican levels are increased [5,174,175]. In the circulation, increased levels of endostatin, matrikine of type XVIII collagen α1, are found, which correlates with disease severity and survival [5,41]. In ECs of PAH patients, COL4A1 and COL4A2 are reduced [173]. COL4A1 and COL4A2 are downstream targets of BMPR2, as reduced levels of BMPR2 and β-catenin lead to decreased COL4A1 and COL4A2 expression, resulting in impaired migration and adhesion [173,176]. The culture of PAEC on type IV collagen and laminin strengthen barrier and integrity. While adhesion to type IV collagen triggers nuclear translocation of YAP/TAZ, adhesion to laminin α5β2γ1 retains YAP/TAZ in the cytoplasm [4]. Endostatin blocks ID-1 gene expression and decreases THBS1-transcription and production [177]. In return, TSP-1 inhibits pulmonary EC proliferation and migration via CD36/CD47, suggesting an endostatin–ID1–TSP1 axis [177]. IPAH; idiopathic pulmonary arterial hypertension; BM, basement membrane; PAs; pulmonary arteries; COL4A5, collagen type IV alpha 5 chain gene; COL14A1, collagen type XIV alpha 1 chain gene; COL18A1, collagen type XIII alpha 1 chain gene; ECs, endothelial cells; PAH, pulmonary arterial hypertension; BMPR2; bone morphogenetic protein receptor type II; β-catenin, beta catenin; COL4A1, collagen type IV alpha 1 chain gene ; COL4A2, collagen type IV alpha 2 chain gene; PAEC, pulmonary arterial endothelial cell; YAP; Yes-associated protein; TAZ, Tafazzin ; ID1, DNA-binding protein inhibitor ID-1; THBS1, thrombospondin-1 gene; TSP-1, thrombospondin-1,.

Several alterations in the expression of BM components have been identified in PH (see Figure 2). A comparative transcriptomic analysis of healthy and remodeled small, distal PAs obtained via laser-capture microdissection identified the BM as one of the most affected cellular components [4,5], and COL4A5, COL14A1, and COL18A1 were upregulated in small remodeled PAs, in the intima and media layer [5]. Other BM collagens, COL4A1/COL4A2, were decreased in isolated and cultured ECs from IPAH [173]. In larger PAs, BM glycoproteins such as laminins were mostly affected [4]. Similarly, other BM components, such as the proteoglycan versican, have been elevated in remodeled PAs [174], and protein levels of BM HSPGs agrin and perlecan were higher in lung tissue of PAH patients [175]. Ultimately, those changes are reflected on the ultrastructure level where the BM appeared thickened, repeatedly underlined by areas with partially degraded BM-like material, and occasionally multiple-layered in remodeled arteries [4].

### 4.2. BM Components on EC Function in PH

#### 4.2.1. Type IV Collagen and Laminin in PH

Individual BM components can differentially alter PH-related function of PAECs. Seeding on type IV collagen and laminin, respectively, increased PAEC barrier integrity. However, only type IV collagen increased cell adhesion and induced Yes-associated protein (YAP) nuclear translocation (see Figure 2). In contrast, laminin lead to cytoplasmic retainment of YAP [4]. YAP is one of the key components of the mechanosensing machinery, and it is increasingly being connected to PAH pathology [169,178]—for example, by being part of the pro-proliferative Hippo-YAP/Tafazzin- integrin-linked kinase 1 (TAZ- ILK1) circuit involved in mediating the hyperproliferation of vascular cells in PAH [169,179,180].

Type IV collagen α1 and α2 have also been linked to bone morphogenetic protein receptor type II (BMPR2) signaling, which is a key molecule in PAH pathology. BMPR2 is a member of the TGF-β superfamily, and its mutations account for approximately 80% of all heritable and 25% of all idiopathic PAH cases [181,182]. The knockdown of BMPR2 in the endothelium concomitantly reduced COL4A1 and COL4A2 expression, which lead to endothelial dysfunction including reduced adhesion, migration, and tube formation [173,183] (see Figure 2). A similar phenotype was observed upon the silencing of β-catenin, which is a downstream mediator of BMPR2 signaling [173]. In line, decreased EC barrier integrity and the formation of tight junctions after the silencing of another type IV collagen, COL4A5, were reported; however, a link to BMPR2 was not yet examined [4].

#### 4.2.2. Type XVIII Collagen and Endostatin in PH

In PAH, increased gene and protein expression of COL18A1 and its proteolytic fragment, endostatin, have been described in remodeled vessels [4,5], and elevated levels of endostatin have been reported in the plasma of PAH patients in several independent patient cohorts [5,40,41]. Plasma levels correlated with worsened cardiac function and poor patient survival [5,41], suggesting endostatin as a biomarker or even predictor for therapy success. Further investigations revealed that within the PAH patient group, an increased occurrence of a mutated version of COL18A1 (in the endostatin region) was observed [41]. This missense variant leads to the incorporation of asparagine (N), instead of the ancestral aspartic acid (D) at residue 104 of endostatin (see Table 4). Although the general PAH population showed increased endostatin plasma levels, the mutation carriers (N/D104ES) had significantly lower endostatin levels and better survival then non-mutation carriers (D/D104ES) [41]. The intriguing fact that PAH patients have increased endostatin levels, yet simultaneously are more likely to carry a protective missense mutation leading to lower endostatin levels, highlights the need for further in-depth investigation of the causative and functional role of endostatin.

To gain functional insight, full-length and short fragments of endostatin, ES_99–111_N104 (mutated) and ES_99–111_D104 (non-mutated), were investigated [177]. The mutated peptide was less potent in inhibition of endothelial cell migration compared to non-mutated peptide and full-length endostatin, further supporting the protective phenotype of the mutated variant in PAH patients [177]. However, no effect of either of the short peptides was found on proliferation, suggesting that different regions within endostatin, or the full-length version, interfere with proliferative processes. Full-length endostatin exerted its effects on apoptosis and migration via inhibitor of DNA binding 1/thrombospin-1 (ID-1/TSP1) axis [177] (see Figure 2).

Indeed, the role of endostatin in PAH pathology might encompass an even wider range of pathways, as suggested by the plethora of reports on its function on endothelial cells. By direct binding to integrin α5β1 and caveolin-1 [129,164] and inhibition of VEGF effects as an antagonist on VEGFR2 (KDR/Flk) [130], endostatin disrupts the cytoskeletal rearrangement and the focal adhesion complex via the inhibition of paxillin and FAK phosphorylation [185]. Both paxillin and FAK signaling at the focal adhesion machinery are involved in mediating some of the phenotypic alteration in pulmonary arterial smooth muscle cell behavior in PAH, such as hyperproliferation, migration, and apoptosis resistance [186,187,188,189].

One of the endostatin binding partners is caveolin-1, which lines the inner side of caveolae conferring their structure [190]. In EC, caveolae functions as a hub for signal transduction and information trafficking. The disruption and damage of EC lead to a loss of caveolin-1 and subsequent barrier disruption [191]. In addition, caveolin-1 can regulate the vascular tone. A loss of caveolin-1 results in impaired store-operated Ca^2+^ entry, leading to reduced synthesis of vasodilatory prostacyclin [192] as well as endothelium-derived hyperpolarizing factor [193]. In PAH patients, a reduction of caveolin-1 has been reported [194,195] and a *CAV1* (gene encoding for caveolin-1) mutation is associated with PAH [196]. Caveolin-1 is pivotal for nitric oxide (NO) production [197]. Currently, all medications for PAH target vasoconstriction. Therefore, the interaction of endostatin with caveolin-1 in EC might potentially interfere with vasoconstriction in PAH. Indeed, studies from the systemic circulation using vascular rings [198] and in vivo studies report endostatin mediated vasorelaxation via NO production [199]. This was supported by in vitro studies, documenting an endothelial nitric oxide synthase (eNOS)-dependent release of NO from EC following endostatin treatment [200].

In addition to vasorelaxation, two independent studies revealed that caveolin-1 [201] and endostatin [158,202] independently inhibit canonical WNT signaling in EC of systemic origin. WNT is a developmental pathway, consisting of a canonical (WNT/β-catenin) and non-canonical (WNT/PCP or WNT/Ca^2+^) pathway [203]. In IPAH, both pathways are upregulated [176,204,205] and can lead to PAEC proliferation, survival, and migration [176]. In EC of systemic origin, endostatin potently reduced the canonical pathway and induced autophagy via Beclin-1 [158]. Whether and how endostatin is involved with alteration in either canonical or non-canonical WNT signaling in PAH has not been investigated.

All together, these data highlight the potential of endostatin to interfere with PAH pathology. However, its controversial functions, from pro-apoptotic to vasodilatory also pinpoint the need of future conclusive in vivo and ex vivo experiments.

#### 4.2.3. BM HSPGs in PH

In addition to BM collagens, also other BM components have been associated with PH, including the two major BM HSPGs, agrin and perlecan. In an unbiased proteomic analysis of lung tissue, the protein cores of agrin and perlecan were identified as upregulated in IPAH compared to donors [175]. These two HSPG differ in regard to their localization: agrin was predominantly found in the subendothelial BM and perlecan was found in the BM underneath endothelial cells and around smooth muscle cells [4,206]. On the structural cells of the vessel wall, perlecan with its heparan sulfate chains may exert different functions, as it is reported to induce angiogenesis [207], yet to inhibit smooth muscle cell proliferation [208,209]. Additionally, PDGF-BB, one of the major factors driving vascular remodeling, contains a retention motif that allows binding to perlecan, thereby establishing a growth factor/morphogen gradient [210,211]. Given this complex role, in vivo studies were needed to clarify its function in PH. Perlecan-HS (Hspg2(Δ3/Δ3))-deficient mice had signs of pulmonary vascular abnormalities already when kept in normoxic conditions, with fewer pericytes and the reduced muscularization of intra-acinar vessels. When challenged with hypoxia to induce PH, perlecan–HS mutant mice showed reduced right ventricular hypertrophy, right ventricular systolic pressure, and vessel muscularization. The protective phenotype was multifactorial, including altered cell–matrix interaction (increased adhesion of SMC to fibronectin), reduced SMC proliferation, and reduced bioavailability of tethered growth factors [212]. Perlecan also influenced the regulation of the vascular tone, as perlecan deficiency reduced eNOS expression in aortas [213].

The proteolytic digestion of perlecan leads to the release of endorepellin from its C-terminal domain. Similar to endostatin, it can be liberated from its parent molecule by the proteolytic enzyme cathepsin L [122] (see Table 2). Increased expression of cathepsin L was found in the endothelium of IPAH patients with BMPR2 mutations and in rat exposed to hypoxia, and genetic ablation of cathepsin L prevented experimental PH [214]. Interestingly, it has been shown that pro-apoptotic caspase 3 activation can induce the secretion of cathepsin L from endothelial cells [122]. Whether this mechanism is involved in PAH and leads to increased endorepellin (and/or endostatin) levels and thus apoptosis of EC has not been examined so far.

Although the higher abundance of the second most common BM HSPG, agrin, has been reported in PH several years ago [175], its functional role in the pulmonary circulation was not investigated. However, studies from other fields have reported that agrin is capable of activating the mechanosensitive YAP signaling of the Hippo pathway [215], suggesting that increased levels of agrin might further propagate pathological cell behavior in PH. Versican is a constituent of the BM, but it is not BM restricted. Similarly, to HSPGs, it is also part of the cell surface glycocalyx [216]. Its expression is usually rather low, but it often increases dramatically in disease. Indeed, in PH, versican accumulates in the vascular lesions in the media, neointima, and plexiform lesions [174]. Whole-exome sequencing revealed that VCAN (gene encoding for versican) is frequently mutated in PAH patients [184], implying a possible function in development of PH, which needs to be verified in future studies.

#### 4.2.4. Inflammation in BM Degradation in PH

In the last years, the role of inflammation and inflammatory processes in PH has increasingly gained attention. Inflammatory cells are found in an increased abundancy in remodeled vessels [217,218,219] and their numbers correlate with the degree of remodeling [217], suggesting that inflammatory cells play an active role in the disease. Inflammatory cells are proposed to be one of the main sources of proteolytic enzymes, such as MMPs or serine elastases, and therefore are capable of modulating ECM remodeling in their proximity [220].

Numerous studies have investigated the role of matrix degrading proteases and their inhibitors in detail [99] and the altered expression and activity of MMPs [167] (MMP-2 [221,222], -7 [223], -9,-10 [5], -19 [5]), ADAMs (ADAM17, ADAM33) [5], cathepsins (Cath S [224], Cath L [214]) and TIMPs (TIMP1 and TIMP3) [5] has been strongly connected with vascular and ECM remodeling. Several of those enzymes are highly abundant in certain inflammatory cells such as monocytes, macrophages, neutrophils, or mast cells and are often upregulated upon immune cell activation [220]. Mast cells, in addition to MMPs [225] and cathepsins [226], secrete another unique set of proteases, including tryptase [227] and chymase [228,229], which in turn can activate MMPs that are often involved in BM degradation, such as MMP-9 [230]. Although MMP-9 is the most extensively studied BM degrading enzyme, many more are involved in BM degradation and the release of matrikines. A summary of enzyme leading to matrikine release from the individual BM components is provided in Table 2. Except for endostatin, BM-derived matrikines have not been intensively studied in the pulmonary circulation. Given the plethora of BM degrading enzymes involved in PH and the fact that immune cells are the major source of MMPs, it is likely that other matrikines, except for endostatin, are also liberated and may have an active role in PH.

Additionally, transendothelial immune cell migration might further propagate the disruption of the BM and the subsequent release of other BM-derived matrikines. Indeed, endothelial cells in PAH are characterized by a hyperinflammatory phenotype with increased nuclear factor kappa-light-chain-enhancer of activated B cells (NF-ĸB) signaling [231,232], which can contribute to immune cell recruitment. One major factor that activates NF-ĸB signaling [233] and primes EC for immune cell transmigration is TNF-α [10]. In line, PAH patients have increased levels of circulating TNF-α correlating with poor survival [234]. Furthermore, matrikine production can be increased upon tissue damage following an insult or injury. Exemplarily, type IV collagen matrikines arresten and canstatin are increasingly released by cathepsin S at the site of injury after myocardial infarction [110].

## 5. Future Perspectives

An “inflamed” endothelium, a shift in immune cell abundancies, and changes in both BM levels and its degrading enzymes point toward a role of BM matrikines in the propagation of endothelial dysfunction in PAH. Therefore, targeting matrikines and degrading enzymes could potentially lead to the development of novel therapeutic strategies for PAH. However, a deeper understanding of how matrikines are released and can influence endothelial function is needed. Deciphering the complexity underlying the cause of matrikine release and identifying BM matrikine-receptor/binding partners and their downstream signaling might pave the way to developing new therapeutics diminishing endothelial dysfunction and thus vascular remodeling. In future, translational studies will hopefully shed more light on this open field of investigation in PH.

## Figures and Tables

**Table 1:** List of major BM components and their function within the BM.

BM Component	Function in BM
BM Glycoprotein	
Laminin	Assembly
Nidogen-1	Assembly and Integrity
Nidogen-2	Integrity
BM Type IV Collagens	
Type IV Collagen heterotrimers	Integrity and Maintenance
BM HSPGs	
Perlecan	Integrity and Stability
Type XVIII Collagen α1	Integrity and Stability
Agrin	Integrity and Stability

BM, basement membrane; BM HSPGs, basement membrane heparan sulfate proteoglycans.

**Table 2:** BM components and their corresponding matrikines.

Parent Protein	BM Matrikine	Proteolytic Enzyme	Molecular Weight (kDa)	Receptor on ECs
BM Glycoproteins				
Laminin α5	AQARSAASKVKVSMKF [105]	n/a	n/a	n/a
Nidogen-1	G3 Domain	MMP-19 [106] CathepsinS [107] Mephrin-α [108]	90	n/a
BM Type IV Collagens				
α1 chain	Arresten [109]	Cathepsin S [110] MMP-14 MMP-15 [111]	26	α1β1 integrin [102]
α2 chain	Canstatin [112]	Cathepsin S [110] MMP-14 MMP-15 [111]	24	αVβ1 integrin [113] αVβ3 αVβ5 integrins [113,114]
α3 chain	Tumstatin [115,116]	MMP-9 [116]	28	αVβ3 αVβ5 integrins [56,113]
α4 chain	Tetrastatin (α4(IV)NC1 domain) [117]	n/a	28	n/a
	Tetrastatins [118]	n/a	~2	n/a
α5 chain	Lamstatin (α5(IV)NC1) [119]	n/a	25	n/a
	Pentastatin [118]		~2	β1 and β3 integrins [120]
α6 chain	α6(IV)NC 1 domain [103]	n/a	25	αVβ3 integrin [113]
	Hexastatin [118]	n/a	~2	n/a
BM HSPGs				
Perlecan	Endorepellin [121]	Cathepsin L [122]	81	α2β1 integrin [123,124] VEGFR2 [124]
	Endorepellin LG3 Domain [125]	BMP1/TLD-like protease [125] Cathepsin L [122] t-PA [122]	23	n/a
Type XVIII Collagen α1	Endostatin (ES) [126]	Elastase [127] Cathepsin L Cathepsin B Cathepsin K Cathepsin S Cathepsin D [128] MMP-3 MMP-9 MMP-12 MMP-13 MMP-20 [126]	20	α5β1 integrins [129] Caveolin-1 [129] VEGFR2 [130] Glypican 1/2 [131] Nucleolin [132]
	Neostatin 7 [133]	MMP-7 [133]	28	n/a
	Neostatin 14 [134]	MMP-14 [134]	28	n/a
Agrin	C-Terminal Agrin Fragment [135]	Neurotrypsin [135]	22	n/a
BM CSPGs				
Versican	Versikine [136]	ADAMTS [136,137]	49	n/a

G3 domain, globular 3 domain; Endorepellin LG3 domain, Endorepellin laminin-like globular 3 domain; MMP, matrix metalloproteinase; VEGFR2, vascular endothelial growth factor tyrosine kinase receptor 2; t-PA, tissue-type plasminogen activator; BMP1/TLD-like protease, bone morphogenetic protein 1/tolloid-like protease; CSPGs, chondroitin sulfate proteoglycans; TLR2, Toll-like receptor.

**Table 3:** Matrikine action on endothelial cells.

Matrikine	Anti-Angiogenic	Anti-Migratory	Anti-Proliferative	Pro-Apoptotic	Actin Disassembly
BM-Type IV Collagen					
Arresten	+	+	+	+	−
Canstatin	+	+	+	+	−
Tumstatin	+	+	+	+	−
α4 NC1	−	+	+	n/a	−
α5 NC1	−	+	+	n/a	−
α6 NC1	+	+	+	n/a	−
Tetrastatin	−	+	+	n/a	−
Pentastatin	−	+	+	n/a	−
Hexastatin	**−**	**+**	+	n/a	−
BM HSPGs					
Endostatin	+	+	+	+	+
Endorepellin	+	+	+	+	+

NC, non-collagenous; BM, basement membrane; BM HSPGs, basement membrane heparan sulfate proteoglycans.

**Table 4:** Identified BM gene mutations in IPAH patients.

Gene	Mutation Detection Method	Variant	Amino Acid [Codon]	Reference
COL18A1	WGS	rs12483377	D [GAC] > N [AAC]	[41]
VCAN	WES	NS	NS	[184]

COL18A1: type XVII collagen alpha 1; VCAN, versican; WGS, whole genome sequencing; WES, whole exome sequencing; ES, endostatin; NS, not shown.
